# Impact of Skin Disinfection on Cutaneous Microbiota, before and after Peripheral Venous Catheter Insertion

**DOI:** 10.3390/antibiotics11091209

**Published:** 2022-09-07

**Authors:** Manon Prat, Jeremy Guenezan, Bertrand Drugeon, Christophe Burucoa, Olivier Mimoz, Maxime Pichon

**Affiliations:** 1 CHU Poitiers, Bacteriology Laboratory, Infectious Agents Department, 86021 Poitiers, France; 2 INSERM U1070, Pharmacology of Antimicrobial Agents and Antibiotic Resistance, University of Poitiers, 86073 Poitiers, France; 3 CHU Poitiers, Emergency Room Department, 86021 Poitiers, France

**Keywords:** peripheral venous catheter, prevention, antiseptics, skin microbiota, sequencing

## Abstract

Introduction. Patients with invasive medical devices are at high risk for infection. Skin colonization is the initial stage of these infections, leading to the recommendation of practices requiring disinfection using antiseptics. Microbial communities playing a major role in skin health could be impacted by antiseptic procedures. Aim. To characterize and compare the bacterial communities of skin samples from patients before an antisepsis procedure, and after removal of the medical device itself, according to the nature of the antiseptic molecule (povidone iodine or chlorhexidine). Methods. The study focused on alterations in bacterial communities depending on the nature of the antiseptic procedure and type of intravascular device. After amplification of 16S rDNA, libraries (*n* = 498 samples) were sequenced using MiSeq platform. Results. Using an in-house pipeline (QIIME2 modules), while no alteration in skin microbiota diversity was associated with antiseptic procedure or PVC type, according to culture results (*p* < 0.05), alterations were at times associated with restricted diversity and higher dissimilarity (*p* < 0.05). Antiseptic procedures and PVC types were associated with the modification of specific bacterial representations with modulation of the Bacillota/Bacteroidota (Firmicutes/Bacteroidetes) ratio (modulation of *C. acnes*, *Prevotella*, *Lagierella*, and *Actinomyces* spp.) (*p* < 0.05). At baseline, the microbiota shows certain bacteria that are significantly associated with future PVC colonization and/or bacteremia (*p* < 0.05). All of these modulations were associated with altered expression of metabolic pathways (*p* < 0.05). Discussion. Finally, this work highlights the need to optimize the management of patients requiring intravascular devices, possibly by modulating the skin microbiota.

## 1. Introduction

The skin is a very particular habitat, colonized by a very unique set of micro-organisms [1]. Microbial communities play a major role in skin health and disease, including immune maturation and resistance to colonization by skin pathogenic microorganisms. [2,3,4]. As in other anatomical niches, previous studies have described a very stable community, which varies not only between individuals but also within a particular individual [5,6]. The most obvious intervention that could disrupt the integrity and function of the microbiota is based on the administration of antimicrobials to reduce the risk or impact of infection by microorganisms that, while not focused on this microbiota itself, could impact the skin microbiota [7,8]. In the first place, this is particularly important for antiseptic molecules (or mixtures), i.e., antimicrobial agents used for their non-specific mechanisms of action [9,10].

Healthcare-associated infections can be responsible for significant morbidity and mortality, but this varies by type of healthcare facility. Patients with invasive medical devices, such as central or peripheral venous catheters (CVCs), are at high risk for infection because of their open access point to the bloodstream [11]. Colonization of the skin by potentially pathogenic microbial strains is the initial stage of these infections, which leads to the recommendation of practices requiring skin disinfection with antiseptic mixtures. As previously mentioned, these molecules, which have non-specific mechanisms of action, have a broad-spectrum impact on the microbiota, particularly on the cutaneous microbiota [12,13,14,15]. The latter has an important role in reducing the risk of infection, justifying the need not to alter the homeostasis of the skin microbiota (for example, by selecting certain species, including environmental germs, which are resistant to the antiseptic procedures used). Although antiseptic procedures have been extensively studied in the short term, little is known about the impact of antisepsis on the skin microbiota, particularly in relation to the molecule and the clinical outcome [12,13,16,17].

In this context, we performed a microbiological study of a real-life clinical study to describe the change in skin microbiota during hospitalization of patients requiring a peripheral venous catheter (PVC), randomized according to the nature of the antiseptic agents.

## 2. Results

### 2.1. Study Population

During the inclusion period, 166 patients met all the inclusion criteria and none of the exclusion criteria and were successfully included. Baseline characteristics are summarized in Table 1. No difference could be observed between groups in terms of baseline characteristics and time between catheter insertion and removal. One may note over-representation of samples ≥10^3^ UFC/mL if culture-positive in the povidone-iodine alcohol group compared to the chlorhexidine alcohol group.

### 2.2. No Alteration in the Diversity of the Skin Microbiota Was Associated with Antiseptic Procedure or Type of PVC, According to the Results of Blood or PVC Cultures

**Figure 1:**
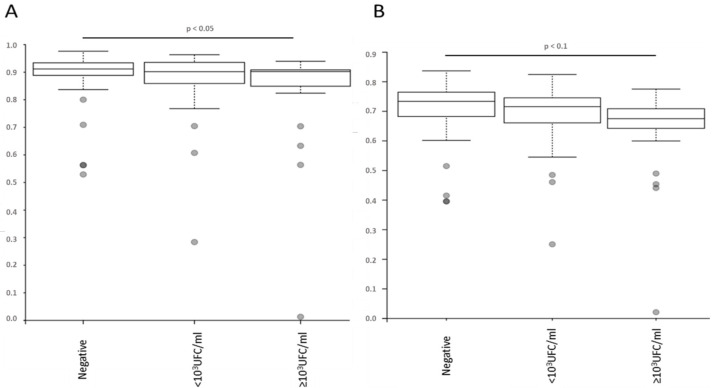
Comparison of alpha-diversities, determined with different indexes ((**A**) Simpson; (**B**) Pielou) according to the results of the PVC culture. The box plots represent the mean and interquartile range for each index, and the vertical axis is the respective index value.

Regarding the alpha diversities, no differences could be observed according to sex (*p* > 0.05). On the contrary, differences could be observed (for all indices) according to patient age group, with more differences between the extreme age groups (18–30 and >90 years; *p* < 0.05). While no difference could be observed between the D0 (skin microbiota sampled before antiseptic exposure) and D+ (skin microbiota sampled before PVC removal) samples regarding these diversities (*p* > 0.05), an impact of the time between them, for samples within 24 h of each other, was observed, with more difference for longer duration (*p* < 0.05). No difference in terms of alpha diversity was observed on the D+ samples regarding the nature of the antiseptic procedure or the innovative or usual PVC (*p* > 0.05). No difference in alpha diversity could be observed regarding the positivity of a blood culture during the clinical history of the sampled patient (*p* > 0.05). On the other hand, statistical differences or trends were observed, with a restriction of diversity, concerning the presence/absence of bacteria colonizing the catheter, when ≥10^3^ UFC/mL (Simpson and Pielou index, *p* < 0.05 and *p* < 0.1, respectively) (Figure 1). Of note, the presence of a concurrent non-cutaneous infection did not significantly impact the alpha diversities of the samples (*p* > 0.05).

**Figure 2:**
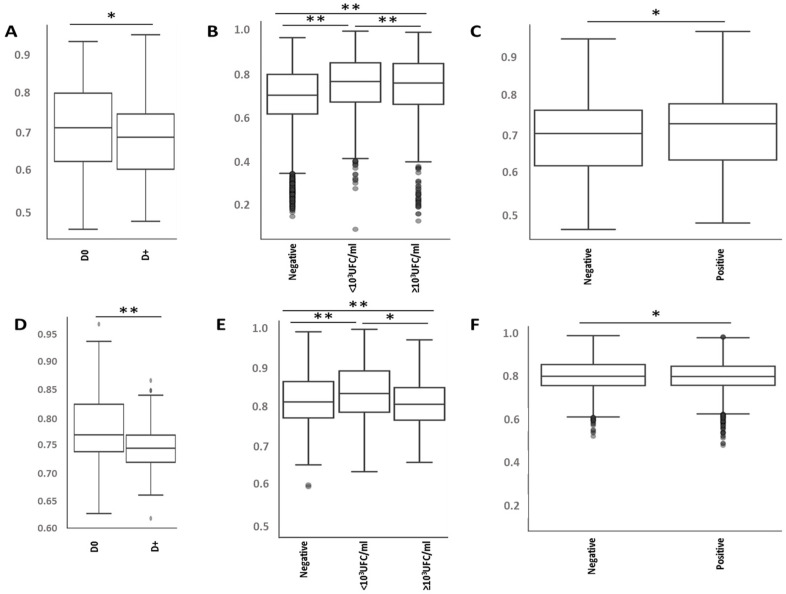
Comparison of beta-diversities, determined with different indexes (Bray Curtis Indexes, (**A**–**C**); and Jaccard indexes, (**D**–**F**)) according to the nature of the sample (D0 or D+; (**A**,**D**)) or results of the PVC (**B**,**E**) and blood cultures (**C**,**F**). *: *p* < 0.05; **: *p* < 0.01; D0: initial sample; D+: ablation sample. The box plots represent the mean and interquartile range for each permutation, and the vertical axis represents the distance between groups.

Regarding beta diversities, a slight difference was observed with respect to sex for the Jaccard and unweighted Unifrac indices (*p* < 0.05) and all indices tested confirmed a difference according to patient age (*p* < 0.05), especially for extreme ages (>90 years). On the other hand, no difference in diversity could be observed with regard to assigned antiseptic procedure and type of PVC (*p* > 0.05). Given that sample diversities were significantly different between the D0 and D+ samples (*p* < 0.05), diversities for all indices were different by blood culture result (sterile or below/above threshold; *p* < 0.05) and by positivity of a blood culture sample (*p* < 0.05, except for Unifrac parameters, which only showed a trend) (Figure 2).

### 2.3. Antiseptic Procedures and PVC Types Are Associated with the Modification of Specific Bacteria Representations

Although microbiota composition analysis did not predict clinical outcome or therapeutic management, all parameters showed a difference in the respective proportions of certain bacteria. Initially, the parameter of age was associated with an overrepresentation of *Cutibacterium* sp. and *Corynebacterium propinquum*/*pseudodiphtericum* in elderly patients (especially after 75 years).

Samples could be distinguished according to their composition. On the phylum level, the Bacillota/Bacteroidota (Firmicutes/Bacteroidetes) ratio was significantly two-fold lower in the D+ sample compared to the D0 sample. Moreover, if proportion of Pseudomonadota (Proteobacteria) increased between the two groups, Actinomycetota (Actinobacteria) demonstrated the opposite. On the genus and species level, only overrepresentation was significantly observed, on *Methylobacterium*, *Microbacterium* sp., *Sphingomonas* sp., *Gordonia* (*polyisoprenivorans*) and *Stenotrophomonas* sp., in D+ samples.

Focusing on D+ samples, *Ruminococcus* sp. and *Prevotella* sp. (*P.bivia* and *P. disiens*) were underrepresented in samples associated with positive blood vials. Samples associated with PVC colonization showed underrepresentation of *Prevotella* sp. (especially *P. bivia*), especially when the concentration was above the threshold.

The proportion of certain transient species was modified according to the nature of the antiseptic procedure, with overrepresentation of *Leptotrichia wadei* and *Fenollaria timonensis* after CHG, whereas PVI led to overrepresentation of *Actinomyces israelii* and *Lagierella massiliensis*. Similarly, the nature of the PVC changed when using innovative PVCs, including the proportion of genera (overrepresentation of *Lachnoanaerobaculum* sp., and *Blastococcus* sp.) and bacterial species observed (overrepresentation of *Actinomyces israelii*, and *Lagierella massiliensis*; and underrepresentation of *Prevotella disiens* and *P. buccalis*).

### 2.4. Antiseptic Procedures and PVC Types Are Associated with Modification of the Predicted Metabolic Pathways

Focusing on D+ samples, using MetaCyc prediction, the L-arginine super-pathway is overexpressed, particularly on its degradation to 4-aminobutanoate and L- ornithine production for patients assigned to CHG and/or with an innovative PVC. This L-arginine pathway is underrepresented in case of PVC colonization beyond the considered threshold.

### 2.5. Bacterial Microbiota before PVC Is Not Predictive of Complications but Is Different in Diversity and Composition

**Figure 3:**
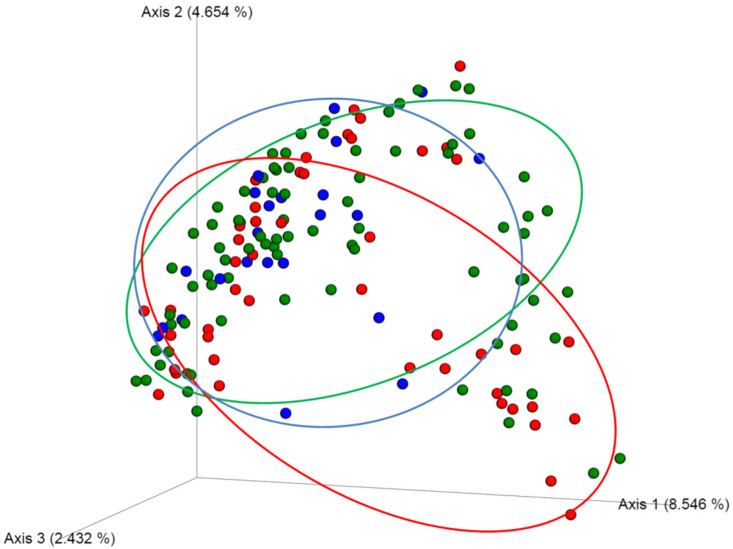
Principal component analysis representation of the skin microbiota at baseline, as a function of the occurrence of PVC colonization. In red and green the samples corresponding to future PVC colonization (above and below the threshold, respectively) are represented. In blue, the samples corresponding to negative culture are represented. PcoA demonstrated that the composition of the skin microbiota at baseline varied according to the colonization status of the PVC at the time of device removal.

Focusing on the D0 samples, no distinction could be observed with respect to clinical parameters (sex and age) or according to randomly assigned groups (antiseptic procedure and PVC), using the alpha diversity indices. For the beta-dissimilarity indices, a statistical difference according to the bacterial concentration colonizing the PVC could be observed on the Jaccard indices, with more dissimilarity between above and below the threshold (*p* < 0.05; Figure 3).

The composition of the microbiota, at baseline, showed certain bacterial genera and species that were significantly associated with future PVC colonization above (*Roseomonas* sp., *Actinomyces* sp., *Corynebacterium kroppenstedtii*, and *Hymenobacter* sp.) or below the threshold (*Eubacterium hallii* and *Ruminococcus* sp.). Similarly, some bacterial taxa were associated with future blood culture positivity when present (*Acinetobacter lwoffii*, *Nesterenkonia* sp., *Escherichia* sp., *Eubacterium hallii*, *Pseudoglutamicibacter* sp., *Flavobacterium* sp., and *Collinsella* sp.) or absent (*Aggregatibacter* sp., *Porphyromonas* sp., *Prevotella* sp. and *Tetragenococcus* sp.). Note that MetaCyc predicted that all these bacteria were implicated in overrepresentation of benzoyl-CoA degradation.

## 3. Discussion

This study is the first to investigate the impact on the skin microbiota of two major antiseptic mixtures (povidone-iodine and chlorhexidine) for skin disinfection before peripheral venous catheters insertion to prevent catheter infection.

Studies on the microbiota have introduced the concept of “dysbiosis”, which can be defined as “possible imbalance with respect to a normal state” [18]. This imbalance could result in a change in the composition or abundance of the entire microbial community or of specific microorganisms. Limiting the validity of Koch’s postulate (indicating that a pathogen causes a distinct disease), they underline the need for a comprehensive and complex interpretation (coupled with thorough understanding) of the interactions between the host, microbial communities, and invasive pathogens.

Since the present study analyzed patients with similar clinical characteristics at the time of inclusion, the dysbiosis observed is solely due to the antiseptic mixture tested. If no difference in terms of alpha diversity (even with lower dissimilarity for ablation samples compared to initial samples) was observed according to the antiseptic procedure used or due to the occurrence of non-cutaneous infection, it is important to observe the impact of a time lapse of more than one day between the two samples of the skin microbiota. This last point agrees with the longitudinal study of the microbiota of non-diseased patients, which demonstrated a return to baseline in 12 to 24 h depending on the nature of the antiseptic molecule/mixture (ethanol or PVI) [19]. In addition, and as expected, a restriction of diversity was observed for the microbiota of patients presenting significant colonization of the intravascular device, attesting to overrepresentation of the species involved in this colonization, at the expense of the species initially present (reciprocally to the change under antibiotic treatment) [20]. This modification, depending on the biological and/or clinical consequences, is confirmed by the demonstration of lower dissimilarity in skin samples from patients with catheter infection (>10^3^ CFU/mL) or positive blood culture compared with samples without.

In addition, it should be noted that the skin microbiota is an ecological niche that could be easily colonized by bacteria present in the hospital environment, depending on the equipment and care activities of the hospital. Even if the two groups analyzed correspond to patients hospitalized in the same hospital environment (monocentric study) and present similar demographic characteristics after selection, justifying that the present study could provide important information on the impact of the antiseptic process on the skin microbiota, very complex interactions could be present and cause the observed discrepancies. This last point underlines the need for further studies to demonstrate the extrapolability of the results obtained.

If the skin microbiota of patients is dominated by *Staphylococcaceae*, it is important to keep in mind that differences in the representation of certain species are found depending on the antiseptic procedure used, attesting to the dysbiosis generated. Post-exposure samples to antiseptic procedures showed a change in their Bacillota/Bacteroidota (Firmicutes/Bacteroidetes) ratio, resulting in increased Pseudomonadota (Proteobacteria) and decreased Actinomycetota (Actinobacteria). In the homeostasis of the skin microbiota, the most recent studies have associated these bacteria with the skin microbiota of prepubertal children, which changes during puberty, without knowing whether puberty allows the acquisition of new species or rather a modulation of those present, leading to inversion of this ratio [21,22]. The Actinomycetota (Actinobacteria), representing between one-third and one-half of the isolated species, mainly composed of *Corynebacterium* and *Cutibacterium*, could also attest to the strong impact of the antiseptic mixture used, with limitation of chlorhexidine to these two bacteria [23,24,25,26]. In addition, it is crucial to understand that the latter two play a major role in controlling the pathogenic strain so as to avoid invasion of wounds or intravascular devices, and the impact on them of antiseptic procedure dysbiosis [27]. The impact of the antiseptic mixture was associated with different overrepresentation of four bacteria, namely *Leptotrichia wadei* and *Fenollaria timonensis* (for chlorhexidine) and *Actinomyces israelii* and *Lagierella massiliensis* (for povidone iodine). To date, the bacteria overrepresented after exposure to CHG have not been associated with modulation of the skin microbiota in health and disease, as they were isolated in oral or stool microbiota alone [28,29]. In contrast, if *L. massiliensis* has been isolated only in stool microbiota samples, *A. Israelii*, which is overrepresented after disinfection with PVI, was associated with skin injuries (described as responsible for 85% of extracervical skin injuries due to *Actinomyces* spp.) [30] Finally, and to a greater extent than the molecule, the overall infusion procedure is associated with modulation of the bacteria present in the physiologically “normal” microbiota. Innovative PVCs, requiring less intervention, have more *Lachnoanaerobaculum* sp. and *Blastococcus* sp. but fewer *Prevotella* sp. (including *P. buccalis* and *P. disiens*). Similarly, the latter were associated with higher concentration of bacterial colonizing PVCs but not with positive blood vials, indicative of bacteremia. Although CVCs with antimicrobial features have been associated with decreased catheter-related colonization and bacteremia, some studies have shown that infection rates may depend more on non-catheter-related factors, such as adherence to infection control standards, choice of insertion site, duration of catheter placement, and frequency of dressing changes. This last point may explain the advantage of using this innovative type of catheter, which limits intervention on the device and prolongs complication-free life [31].

When studying the predicted changes in metabolic pathways, depending on the changes in the species present, a change in the arginine catalytic pathway after exposure to chlorhexidine appears. In the literature, this pathway has been associated with *Cutibacterium* metabolism, which has demonstrated an ability to grow in the anoxic sebaceous gland, using proteases to release the amino acid arginine from skin proteins and lipases to degrade triglyceride lipids in the sebum, producing free fatty acids that promote bacterial adhesion [32].

The choice of product for rapid and effective skin antisepsis is most often limited to iodine derivatives or alcoholic chlorhexidine, which have demonstrated their efficacy in vitro and in vivo, compared to other molecules such as sodium hypochlorite or ethanol alone [14,15,33,34]. Nevertheless, a detailed study of human bacteriome has shown that recolonization of the skin is an inexorable effect, whatever the antiseptic procedure [35]. Furthermore, and by design, 16S metabarcoding approaches detect resident as well as transient bacteria (including “pathogenic” and “non-pathogenic” bacteria) in the skin microbiota. Nevertheless, highlighting bacteria of low pathogenicity and transient presence remains of important interest on two axes. First, from a clinical standpoint, and even if it does not result in bacteremia or other serious infection, local infection/inflammation caused by these bacteria could be detrimental to the patient, especially in hospitalized patients, and could result in (costly) prolonged hospitalization or morbidity [36,37,38]. Secondly, from a microbiological point of view, it has been demonstrated using co-culture protocols that cooperation between bacteria can result in a different pathogenicity [39]. Given this fact, even “non-pathogenic” bacteria (without any described pathogenicity at the moment), must be identified to understand the pathogenicity of all bacteria in these medical contexts.

This study has some limitations. First, the choice of sampling site, justified by usual patient management for PVC use in the hospital setting, could not be generalized to the whole body, as bacterial distribution is known to vary across the body [40]. Some studies have shown that the kinetics of microbiota restoration are to some extent a function of the location of the harvested anatomical site [19,22]. More than just a temporal consideration, as the core microbiota is different from site to site, it is obvious that antiseptic procedure septic impacts also differ. Second, the included population was elderly and had potentially confounding comorbidities or diseases, which limits generalization to a younger and healthier population, but did not impact the quality of comparisons between antiseptic protocols through initial randomization. Previous studies have shown that the composition of the human skin microbiota varies profoundly with age [41]. Nevertheless, it is important to note that, despite the constant disruptions that human skin undergoes in daily life, healthy adults stably maintain their skin communities for at least two years, while the dominant characteristics of skin microbial communities remain stable indefinitely, which is similar to the stability observed in the gut that allowed the comparison, in younger and older patients alike [22].

## 4. Materials and Methods

### 4.1. Selected Samples

The present study was an ancillary to the CLEAN3 study [14]. Briefly, CLEAN 3 is an investigator-initiated, open-label, single-center, randomized, two-by-two factorial trial. Patients were prospectively recruited in the emergency department of the Poitiers University Hospital in France between 7 January 2019 and 6 September 2019. The objectives of the CLEAN3 study were to analyze the clinical impact of antiseptic agents (2% chlorhexidine-alcohol, CHG, or 5% povidone iodine-alcohol, PVI) and the nature of the intravascular devices employed (standard-of care or innovative).

Patients were included if they fulfilled all the following criteria: (i) adult patients (≥18 years); (ii) requiring hospital admission in medical wards and placement of a single PVC for 48 h or longer; (iii) obtained written informed consent. Were excluded patients who presented at least one of the following criteria: (i) known intolerance, hypersensitivity or contraindication to any trial drug; (ii) being suspected of having a bloodstream infection; (iii) having skin injury increasing the risk of catheter infection; (iv) having intravascular catheter in place within the last 2 days, or within the last 2 weeks and with local signs of catheter complication; (v) being suspected of difficult catheter insertion (obesity, known IV drug users, non-visible venous network after placement of a tourniquet…); (vi) requiring surgery; (vii) having previously been enrolled in this study. All included patients were centrally randomized for antiseptic procedure allocation and type of PVC.

In addition to the CLEAN3 selection criteria, the following exclusion criteria were added due to their impact on the skin microbiota: (i) immunosuppression status (autoimmune disease, immunosuppressive treatment or disease); (ii) antibiotic treatment within the last two weeks before inclusion [42].

All included patients were sampled twice (before placement and after removal of the PVC) from the same anatomic region according to the protocol described in the Manual of Procedures for the NIH Human Microbiota Project [35,43]. Before disinfection according to the allocated protocol, gentle dermabrasion of the skin (5 cm × 5 cm square on the antecubital region of the perfused arm) was applied with an appropriate swab and then poured into nucleic acid preservative medium (eNat, Copan, Italy), before being stored at −80 °C until analysis (sample “D0”). Similarly, the same anatomic region was sampled after catheter removal (Sample “D+”).

Culture of PVC tips was performed according to the 2018 recommendations of the French Society of Microbiology EUCAST (SFM). Briefly, intravascular PVC tips were collected in a sterile vial and mixed with 1 mL of saline. After plating 100 µL of this saline on blood agar for 48 h at 35+/−2 °C, all growing bacteria were identified using a MALDI-TOF (VitekMS, bioMérieux, Marcy l’étoile, France) and enumerated to distinguish colonized (according to the threshold of 10^3^ Colony Forming Units, CFU, per mL) from uncolonized PVCs. In addition to the volume used for routine diagnosis, 200 μL of saline samples used for vortex of PVCs were mixed with RNAlater (Life Technologies, Carlsbad, CA, USA) (ratio 1:2) in a LowBinding tube (Eppendorf, Hamburg, Germany) before being frozen and stored at −80 °C until analysis [14].

Note that all relevant clinical information was collected prospectively during sample collection by the investigators involved in the study (J.G., B.D., O.M., and M.Pi.). Relevant clinical information could have been considered as potential confounding biases (gender, age, “other” nonvascular infection, time between catheter insertion and removal) or biologically and/or clinically relevant to analyze local inflammation, type of CVP, results of CVP tip culture (routinely obtained), and results of blood culture (if collected during the patient’s routine clinical management).

### 4.2. Sample Preparation

Samples were prepared according to a complete 16S rDNA sequencing in-house protocol [44]. DNA extraction was performed according to manufacturer’s recommendations using an QIAmp^®^ PowerFecal^®^ Pro DNA kit (Qiagen, Venlo, The Netherlands) with association of bead-beating and enzymatic lysis. Nucleic acids were eluted in 50 µL of Low TE buffer (Eurobio, Les Ulis, France) before quantification using the dsDNA High Sensitivity assay (Invitrogen, Life Technologies, Carlsbad, CA, USA) on Qubit 4.0 fluorometer (Invitrogen). According to the quantification, the extract could have to be diluted before initiation of the library production process. A negative control (no-template control) was added to the process and thereafter considered, as were other clinical samples. Analytical positive controls (Skin Microbiota Genomic Mix, ATCC MSA-1005) and ZymoBIOMICS Microbial Community Standard) and negative control (a blank collection/transport medium considered and analyzed like other skin samples) were applied for each experiment to validate the analytical and bioinformatic process and comparisons.

According to Illumina recommendations, libraries were then produced following fusion PCR targeting V1–V3 16S rDNA regions as described previously [23]. Briefly, PCR reactions were carried out at a total volume of 25 μL for each sample, containing final concentrations of 1X KAPA HiFi HotStart ReadyMix (Roche, Bâle, Switzerland), 1 μM 16S-V1-fwd primer, 1 μM 16S-V3-rvs primer, and 100 ng maximum of template DNA per reaction. As in other publications focusing on skin microbiota, PCR was carried out using the following cycling conditions: 95 °C for 3 min; 30 cycles of 95 °C for 30 s, 55 °C for 30 s, and 72 °C for 30 s; 72 °C for 5 min kept at 4 °C; the amplified products were then visualized on a 1.5% agarose gel [45].

After manual verification by two operators (M.Pr. and M.Pi.), validated PCR products corresponding to the D0 and D+ couple of a patient were purified using NucleoMag NGS Clean-up and Size Select beads (Macherey-Nagel, Hoerdt, France) using a 1.8:1 ratio and 80% ethanol as recommended before quantification using the Qubit 1x ds DNA HS Assay Kit (Invitrogen) on the Qubit 4.0 Fluorometer (Invitrogen). Indexing was performed using the Nextera^®^ XT Index Kit V2 Set D (Illumina, CA, USA) according to manufacturer’s recommendations for fusion PCR. After final quantification using the Qubit 4.0 Fluorometer, all libraries were pooled in equimolar amounts for 96-sample total libraries and then diluted to 15 pM, denatured and 5%-PhiX-spiked before final sequencing using a MiSeq V3 paired-end kit.

### 4.3. Bioinformatic Analyses

Microbiota bioinformatics were performed with QIIME 2 2018.8 [46]. After demultiplexing, raw sequence data were trimmed at a length of 280 bp and quality filtered (for an expected error rate of less than 0.1%) using q2-demux followed by denoising using DADA2 [47]. All amplicon sequence variants (ASVs), aligned with mafft, were used to construct phylogeny against the Silva132 99% sequences. [48,49,50]. After rarefaction (subsampling without replacement to 7500 sequences per sample), alpha-diversity metrics (observed, Chao1’s, Shannon’s, Pielou’s indexes and Faith’s Phylogenetic Diversity), and beta-diversity metrics (weighted UniFrac, unweighted UniFrac, and Bray-Curtis dissimilarity) were estimated [51,52,53]. Analysis of the Composition of the Microbiota (ANCOM) and Linear discriminant analysis effect size (LefSe) were used to identify differentially abundant genera/species among groups that were considered if W > 2 and null hypothesis was rejected [54].

Taxonomic relative abundance graphs were prepared, dividing microbiota populations into their respective taxonomic levels [phylum, genus, species]. Data were analyzed using the Fisher exact test or pairwise permANOVA for statistical significance (according to the categorical or continuous nature of the analyzed variable) and reported as follows: *p* ≤ 0.05 (*), ≤0.01 (**), and ≤0.001 (***). Figures were produced using the Qiime2View 2018.8 website. Raw data are available under the Sequence Read Archive BioProject PRJNA6474476 (SRR12276270 to SRR12276516).

### 4.4. Ethical Considerations

The study protocol was approved by the French Southwest and Overseas Ethics Committee and the French Drug Safety Agency, and the study was carried out in accordance with the principles of the Declaration of Helsinki and the Clinical Trials Directive 2001/20/EC and 2005/28/EC of the European Parliament.

## 5. Conclusions

In conclusion, this study calls for clinical studies focusing on the skin microbiota exposed to antiseptic procedure with closer longitudinal sampling during use of intravascular devices. This could provide a more kinetic view of the pharmacodynamics of antiseptic mixture on the skin microbiota. Furthermore, if a sequencing approach enables study of qualitative variations, it is important to consider a quantitative vision of this microbiota, as the impact of the species found is not considered in the same way according to the overall population present. Finally, the approaches used in the present study do not provide information on the presence of antibiotic resistance genes or other specific metabolic pathways stimulated/inhibited by antiseptic exposure. As it evaluated only the effect of acute stressors due to unique perturbations, further research on long-term treatment regimens or continuous exposure to antiseptics mixtures is needed as it is possible that longer-term perturbations could cause even greater changes in human skin bacterial communities. Moreover, it could be of interest to study other antiseptics molecules such as sodium hypochlorite and alcohol alone in the same manner, the objective being to provide a comprehensive overview of the impact of antiseptic procedures on the skin microbiota. Together, these studies will provide a more precise understanding of the mechanisms of action and the role of antiseptics procedure in a context of merging the idea of the need for proper antiseptic mixture management.

## Figures and Tables

**Table 1:** Characteristics of the included patients according to their randomization arm.

Allocation Group		Chlorhexidine-Alcohol (*n* = 76; 45.8%)	Povidone-Iodine-Alcohol (*n* = 90; 54.2%)
Sex (Male; *n*; x% of the respective category)		35 (46.1)	48 (53.3)
Age category in years (*n*; x%)			
	18–30	5 (6.6)	3 (3.3)
	31–45	4 (5.3)	8 (8.9)
	46–60	8 (10.5)	10 (11.1)
	61–75	23 (30.3)	16 (17.8)
	76–90	28 (36.8)	38 (42.2)
	>90	8 (10.5)	15 (16.7)
Innovative PVC type (*n*; %)		35 (46.1)	43 (47.8)
Local inflammation (*n*; x%)		1 (1.3)	-
Positive PVC culture (*n*; %)		33 (43.4)	43 (47.7)
≥10^3^ UFC per mL (*n*; % of the positive culture)		1 (3.0)	23 (53.5)
<10^3^ UFC per mL (*n*; % of the positive culture)		32 (97.0)	20 (46.5)
Positive blood culture (*n*; % of patients sampled for blood culture) *		33 (43.4)	43 (47.8)
Other infection (*n*; %)		3 (3.9)	3 (3.3)
Time elapsed between catheter insertion and removal (mean; SEM)		2.30 (0.23)	2.50 (0.16)

*: all positive blood cultures were considered, including vials contaminated with skin bacteria (coagulase negative *Staphylococci*). Note that the analytical positive controls were valid and that the analytical negative control did not produce sufficient data after applying the filtering parameters described above, thereby validating the following results.

## Data Availability

Raw data are available under the Sequence Read Archive BioProject PRJNA6474476 (SRR12276270 to SRR12276516).

## References

[1] Grice E.A., Segre J.A. (2011). The Skin Microbiome. Nat. Rev. Microbiol..

[2] Nakatsuji T., Chen T.H., Narala S., Chun K.A., Two A.M., Yun T., Shafiq F., Kotol P.F., Bouslimani A., Melnik A.V. (2017). Antimicrobials from Human Skin Commensal Bacteria Protect against Staphylococcus Aureus and Are Deficient in Atopic Dermatitis. Sci. Transl. Med..

[3] Scharschmidt T.C., Vasquez K.S., Pauli M.L., Leitner E.G., Chu K., Truong H.-A., Lowe M.M., Sanchez Rodriguez R., Ali N., Laszik Z.G. (2017). Commensal Microbes and Hair Follicle Morphogenesis Coordinately Drive Treg Migration into Neonatal Skin. Cell Host Microbe.

[4] Zipperer A., Konnerth M.C., Laux C., Berscheid A., Janek D., Weidenmaier C., Burian M., Schilling N.A., Slavetinsky C., Marschal M. (2016). Human Commensals Producing a Novel Antibiotic Impair Pathogen Colonization. Nature.

[5] Costello E.K., Lauber C.L., Hamady M., Fierer N., Gordon J.I., Knight R. (2009). Bacterial Community Variation in Human Body Habitats across Space and Time. Science.

[6] Grice E.A., Kong H.H., Conlan S., Deming C.B., Davis J., Young A.C., Bouffard G.G., Blakesley R.W., Murray P.R., NISC Comparative Sequencing Program (2009). Topographical and Temporal Diversity of the Human Skin Microbiome. Science.

[7] Beausoleil C.M., Paulson D.S., Bogert A., Lewis G.S. (2012). In Vivo Evaluation of the Persistant and Residual Antimicrobial Properties of Three Hand-Scrub and Hand-Rub Regimes in a Simulated Surgical Environment. J. Hosp. Infect..

[8] Carty N., Wibaux A., Ward C., Paulson D.S., Johnson P. (2014). Antimicrobial Activity of a Novel Adhesive Containing Chlorhexidine Gluconate (CHG) against the Resident Microflora in Human Volunteers. J. Antimicrob. Chemother..

[9] Kampf G., Kramer A. (2004). Epidemiologic Background of Hand Hygiene and Evaluation of the Most Important Agents for Scrubs and Rubs. Clin. Microbiol. Rev..

[10] McDonnell G., Russell A.D. (1999). Antiseptics and Disinfectants: Activity, Action, and Resistance. Clin. Microbiol. Rev..

[11] O’Grady N.P., Alexander M., Burns L.A., Dellinger E.P., Garland J., Heard S.O., Lipsett P.A., Masur H., Mermel L.A., Pearson M.L. (2011). Guidelines for the Prevention of Intravascular Catheter-Related Infections. Clin. Infect. Dis..

[12] Huang S.S., Septimus E., Kleinman K., Moody J., Hickok J., Avery T.R., Lankiewicz J., Gombosev A., Terpstra L., Hartford F. (2013). Targeted versus Universal Decolonization to Prevent ICU Infection. N. Engl. J. Med..

[13] Huskins W.C., Huckabee C.M., O’Grady N.P., Murray P., Kopetskie H., Zimmer L., Walker M.E., Sinkowitz-Cochran R.L., Jernigan J.A., Samore M. (2011). Intervention to Reduce Transmission of Resistant Bacteria in Intensive Care. N. Engl. J. Med..

[14] Guenezan J., Marjanovic N., Drugeon B., Neill R.O., Liuu E., Roblot F., Palazzo P., Bironneau V., Prevost F., Paul J. (2021). Chlorhexidine plus Alcohol versus Povidone Iodine plus Alcohol, Combined or Not with Innovative Devices, for Prevention of Short-Term Peripheral Venous Catheter Infection and Failure (CLEAN 3 Study): An Investigator-Initiated, Open-Label, Single Centre, Randomised-Controlled, Two-by-Two Factorial Trial. Lancet Infect. Dis..

[15] Drugeon B., Pichon M., Marjanovic N., Mousse S., Seguin S., Raynaud C., Rahoui A., Frasca D., Mimoz O., Guenezan J. (2021). Peripheral Venous Catheter Colonisation after Skin Disinfection with 0.5% Aqueous Sodium Hypochlorite, Preceded or Not by One Application of 70% Ethanol (DACLEAN): A Single Centre, Randomised, Open-Label, Pilot Study. J. Hosp. Infect..

[16] Lin Z., Farooqui A., Li G., Wong G.K., Mason A.L., Banner D. (2014). Next-Generation Sequencing and Bioinformatic Approaches to Detect and Analyze Influenza Virus in Ferrets. J. Infect. Dev. Ctries..

[17] Wiemken T.L., Ericsson A.C. (2021). Chlorhexidine Gluconate Does Not Result in Epidermal Microbiota Dysbiosis in Healthy Adults. Am. J. Infect. Control.

[18] McLoughlin I.J., Wright E.M., Tagg J.R., Jain R., Hale J.D.F. (2021). Skin Microbiome-The Next Frontier for Probiotic Intervention. Probiotics Antimicrob. Proteins.

[19] SanMiguel A.J., Meisel J.S., Horwinski J., Zheng Q., Bradley C.W., Grice E.A. (2018). Antiseptic Agents Elicit Short-Term, Personalized, and Body Site-Specific Shifts in Resident Skin Bacterial Communities. J. Investig. Dermatol..

[20] Naik S., Bouladoux N., Wilhelm C., Molloy M.J., Salcedo R., Kastenmuller W., Deming C., Quinones M., Koo L., Conlan S. (2012). Compartmentalized Control of Skin Immunity by Resident Commensals. Science.

[21] Oh J., Conlan S., Polley E.C., Segre J.A., Kong H.H. (2012). Shifts in Human Skin and Nares Microbiota of Healthy Children and Adults. Genome Med..

[22] Oh J., Byrd A.L., Park M., Kong H.H., Segre  J.A., NISC Comparative Sequencing Program (2016). Temporal Stability of the Human Skin Microbiome. Cell.

[23] Langevin S., Pichon M., Smith E., Morrison J., Bent Z., Green R., Barker K., Solberg O., Gillet Y., Javouhey E. (2017). Early Nasopharyngeal Microbial Signature Associated with Severe Influenza in Children: A Retrospective Pilot Study. J. Gen. Virol..

[24] Crane J.K., Hohman D.W., Nodzo S.R., Duquin T.R. (2013). Antimicrobial Susceptibility of Propionibacterium Acnes Isolates from Shoulder Surgery. Antimicrob. Agents Chemother..

[25] Lee M.J., Pottinger P.S., Butler-Wu S., Bumgarner R.E., Russ S.M., Matsen F.A. (2014). Propionibacterium Persists in the Skin despite Standard Surgical Preparation. J. Bone Jt. Surg..

[26] Pichon M., Burucoa C., Evplanov V., Favalli F. (2022). Efficacy of Three Povidone Iodine Formulations against Cutibacterium Acnes Assessed through In Vitro Studies: A Preliminary Study. Antibiotics.

[27] Christensen G.J.M., Scholz C.F.P., Enghild J., Rohde H., Kilian M., Thürmer A., Brzuszkiewicz E., Lomholt H.B., Brüggemann H. (2016). Antagonism between Staphylococcus Epidermidis and Propionibacterium Acnes and Its Genomic Basis. BMC Genom..

[28] Lee E., Park S., Um S., Kim S., Lee J., Jang J., Jeong H.-O., Shin J., Kang J., Lee S. (2021). Microbiome of Saliva and Plaque in Children According to Age and Dental Caries Experience. Diagnostics.

[29] Lo C.I., Niang E.H.A., Sarr M., Durand G., Tall M.L., Caputo A., Raoult D., Fournier P.-E., Fenollar F. (2020). Fenollaria Timonensis Sp. Nov., A New Bacterium Isolated from Healthy Human Fresh Stool. Curr. Microbiol..

[30] Machet L., Machet M.C., Estève E., Delarbre J.M., Pelucio-Lopes C., Pruvost F., Lorette G. (1993). Actinomyces meyeri cutaneous actinomycosis with pulmonary localization. Ann. Derm. Venereol..

[31] Moretti E.W., Ofstead C.L., Kristy R.M., Wetzler H.P. (2005). Impact of Central Venous Catheter Type and Methods on Catheter-Related Colonization and Bacteraemia. J. Hosp. Infect..

[32] Holland K.T., Greenman J., Cunliffe W.J. (1979). Growth of Cutaneous Propionibacteria on Synthetic Medium; Growth Yields and Exoenzyme Production. J. Appl. Bacteriol..

[33] Messager S., Goddard P.A., Dettmar P.W., Maillard J.-Y. (2001). Determination of the Antibacterial Efficacy of Several Antiseptics Tested on Skin by an “ex-Vivo” Test. J. Med. Microbiol..

[34] Boisson M., Corbi P., Kerforne T., Camilleri L., Debauchez M., Demondion P., Eljezi V., Flecher E., Lepelletier D., Leprince P. (2019). Multicentre, Open-Label, Randomised, Controlled Clinical Trial Comparing 2% Chlorhexidine-70% Isopropanol and 5% Povidone Iodine-69% Ethanol for Skin Antisepsis in Reducing Surgical-Site Infection after Cardiac Surgery: The CLEAN 2 Study Protocol. BMJ Open.

[35] (2019). Integrative HMP (iHMP) Research Network Consortium The Integrative Human Microbiome Project. Nature.

[36] Rickard C.M., Webster J., Wallis M.C., Marsh N., McGrail M.R., French V., Foster L., Gallagher P., Gowardman J.R., Zhang L. (2012). Routine versus Clinically Indicated Replacement of Peripheral Intravenous Catheters: A Randomised Controlled Equivalence Trial. Lancet.

[37] Tuffaha H.W., Rickard C.M., Webster J., Marsh N., Gordon L., Wallis M., Scuffham P.A. (2014). Cost-Effectiveness Analysis of Clinically Indicated versus Routine Replacement of Peripheral Intravenous Catheters. Appl. Health Econ. Health Policy.

[38] Olivier R.C., Wickman M., Skinner C., Ablir L. (2021). The Impact of Replacing Peripheral Intravenous Catheters When Clinically Indicated on Infection Rate, Nurse Satisfaction, and Costs in CCU, Step-Down, and Oncology Units. Am. J. Infect. Control.

[39] Yung D.B.Y., Sircombe K.J., Pletzer D. (2021). Friends or Enemies? The Complicated Relationship between Pseudomonas Aeruginosa and Staphylococcus Aureus. Mol. Microbiol..

[40] Bouslimani A., Porto C., Rath C.M., Wang M., Guo Y., Gonzalez A., Berg-Lyon D., Ackermann G., Moeller Christensen G.J., Nakatsuji T. (2015). Molecular Cartography of the Human Skin Surface in 3D. Proc. Natl. Acad. Sci. USA.

[41] Ursell L.K., Clemente J.C., Rideout J.R., Gevers D., Caporaso J.G., Knight R. (2012). The Interpersonal and Intrapersonal Diversity of Human-Associated Microbiota in Key Body Sites. J. Allergy Clin. Immunol..

[42] Lloyd-Price J., Abu-Ali G., Huttenhower C. (2016). The Healthy Human Microbiome. Genome Med..

[43] Castelino M., Eyre S., Moat J., Fox G., Martin P., Ho P., Upton M., Barton A. (2017). Optimisation of Methods for Bacterial Skin Microbiome Investigation: Primer Selection and Comparison of the 454 versus MiSeq Platform. BMC Microbiol..

[44] Gachet C., Prat M., Burucoa C., Grivard P., Pichon M. (2022). Spermatic Microbiome Characteristics in Infertile Patients: Impact on Sperm Count, Mobility, and Morphology. J. Clin. Med..

[45] Chang H.-W., Yan D., Singh R., Liu J., Lu X., Ucmak D., Lee K., Afifi L., Fadrosh D., Leech J. (2018). Alteration of the Cutaneous Microbiome in Psoriasis and Potential Role in Th17 Polarization. Microbiome.

[46] Bolyen E., Rideout J.R., Dillon M.R., Bokulich N.A., Abnet C.C., Al-Ghalith G.A., Alexander H., Alm E.J., Arumugam M., Asnicar F. (2019). Reproducible, Interactive, Scalable and Extensible Microbiome Data Science Using QIIME 2. Nat. Biotechnol..

[47] Callahan B.J., McMurdie P.J., Rosen M.J., Han A.W., Johnson A.J.A., Holmes S.P. (2016). DADA2: High-Resolution Sample Inference from Illumina Amplicon Data. Nat. Methods.

[48] Katoh K., Rozewicki J., Yamada K.D. (2019). MAFFT Online Service: Multiple Sequence Alignment, Interactive Sequence Choice and Visualization. Brief. Bioinform..

[49] Katoh K., Misawa K., Kuma K., Miyata T. (2002). MAFFT: A Novel Method for Rapid Multiple Sequence Alignment Based on Fast Fourier Transform. Nucleic Acids Res..

[50] Price M.N., Dehal P.S., Arkin A.P. (2010). FastTree 2--Approximately Maximum-Likelihood Trees for Large Alignments. PLoS ONE.

[51] Faith D.P. (2007). The Role of the Phylogenetic Diversity Measure, PD, in Bio-Informatics: Getting the Definition Right. Evol. Bioinform..

[52] Lozupone C., Knight R. (2005). UniFrac: A New Phylogenetic Method for Comparing Microbial Communities. Appl. Environ. Microbiol..

[53] Lozupone C.A., Hamady M., Kelley S.T., Knight R. (2007). Quantitative and Qualitative Beta Diversity Measures Lead to Different Insights into Factors That Structure Microbial Communities. Appl. Environ. Microbiol..

[54] Mandal S., Van Treuren W., White R.A., Eggesbø M., Knight R., Peddada S.D. (2015). Analysis of Composition of Microbiomes: A Novel Method for Studying Microbial Composition. Microb. Ecol. Health Dis..

